# Therapy Recommendation “Act as Usual” in Patients with Whiplash Injuries QTF I°

**DOI:** 10.5539/gjhs.v4n6p36

**Published:** 2012-08-20

**Authors:** Christoph Dehner, Michael Kraus, Hendrik Schöll, Florian Schneider, Peter Richter, Michael Kramer

**Affiliations:** 1 Department for Trauma, Hand, Plastic and Reconstructive Surgery, University of Ulm, Ulm, Germany; 2 Ulmkolleg School for Physiotherapeuts and Masseurs, Ulm, Germany

**Keywords:** act as usual, therapy recommendation, whiplash injury, QTF classification

## Abstract

Up to now no therapy study has used the classification system of the Quebec Task Force (QTF) to differentiate between patients with (QTF II°) and without functional disorders (QTF I°). This differentiation seems meaningful, as this difference may be relevant for the correct treatment planning. In this context the effect of the therapy recommendation “act as usual” has been evaluated in a homogeneous patient collective with whiplash injuries QTF I°.

470 patients with acute whiplash injuries had been catched in this study and classified according to the QTF. 359 patients (76.4%) with QTF I° injuries could be identified. Out of that 162 patients were enrolled to the study and received the therapy recommendation “act as usual” and the adapted pain treatment with non-steroidal anti-inflammatory drugs (NSAID). After six months the outcome was evaluated by phone.

After injury the median pain score assessed by a visual analogue scale (VAS) was 5.4 (min = 3.3; max = 8.5). After six months 5 of the 162 patients complained intermittent pain symptoms (VAS values < 2). This is consistent with a chronification rate of 3.1%. After injury, the median pain disability index (PDI) was 3.9 (min = 1.9; max = 7.7). After six months 3 of the 162 patients stated persisting disability during sporting and physical activities (VAS values < 1).

The therapy recommendation “act as usual” in combination with an adapted pain treatment is sufficient. Usually patients with whiplash injuries QTF I° do not need physical therapy. An escalation of therapy measures should be reserved to patients with complicated healing processes.

## 1. Introduction

In the age of increasing private transport, one can observe a steady increase in the incidence of whiplash injuries of the cervical spine (Bener, Rahman, & Mitra, 2009; Halpin, Greenspan, Haileyesus, & Annest, 2009; Quinlan, Annest, Myer, Ryan, & Hill, 2004). For example the incidence of whiplash injuries in an economically developed country like Qatar was 2006 calculated with 171/100.000 (Bener et al., 2009). In the U.S. the incidence of whiplash injuries under consideration of principal and secondary injuries was calculated for the year 2004 with 384/100.000 (Halpin et al., 2009). The majority of whiplash injuries of the cervical spine are a domain of conservative treatment. In view of the frequency by which the physicians and physical therapists are confronted with this diagnosis and under consideration of its economic relevance the aim of acute therapy must be the best possible reduction of the healing time and the prevention of chronicity of the symptoms.

With this objective, previous studies have compared a wide variety of therapy measures such as mobilisation (Gross et al., 2004), manual therapy (D’Sylva et al., 2010; Gross et al., 2004), traction (D’Sylva et al., 2010), machine-assisted muscle building training (Taimela, Takala, Asklof, Seppala, & Parviainen, 2000), ultrasound (Koes et al., 1992), electromagnetic waves (Kroeling, Gross, & Houghton, 2005), TENS (Foley Nolan, Barry, Coughlan, O’Connor, & Roden, 1990), nerve stimulation (Provinciali, Baroni, Illuminati, & Ceravolo, 1996) and Caiontophoresis (Provinciali et al., 1996).

While in 1995, the Quebec Task Force (QTF) could not yet find a reliable indication of the superiority of these measures in the treatment of whiplash injuries of the cervical spine as compared to spontaneous recovery (Spitzer et al., 1995), other studies propagate better therapy outcomes in groups with early and intensive physiotherapy (Giebel, Edelmann, & Huser, 1997; Gross et al., 2004; Schnabel et al., 2002). In meta-analyses, these contradictory statements are frequently explained by reference to the poor quality of the conducted therapy studies (Hoving et al., 2001; Peeters, Verhagen, de Bie, & Oostendorp, 2001; Scholten-Peeters et al., 2002).

Only few studies focussing on the therapeutic approach of whiplash injuries give detailed information about the examined study collective (Peeters et al., 2001; Scholten-Peeters et al., 2002). Up to now no therapy study used the classification system of the QTF to differentiate between patients with neck pain without musculoskeletal signs and free range of motion (QTF I°) and patients with neck pain with musculoskeletal signs and restricted range of motion (QTF II°) (Cote et al., 2008; Spitzer et al., 1995). This differentiation seems meaningful as this classification leads to separation of patients with and without functional disorders.

Hartling et al. (2001) could show that the prognosis of the sustained whiplash injury is associated to the initial QTF classification. The higher the initial grade of injury severity, the more likely is the chronification of pain symptoms. Under consideration that up to now only mixed patient collectives with QTF I° and II° injuries had been investigated, it could be expected that the long-time outcome would be better in an isolated patient collective of QTF I° injuries. Furthermore it seems reasonable that the therapeutic necessities of patients with QTF I° injuries may be different from patients with QTF II° injuries. Against this background the following study brings up two questions:

1. In which frequency and relevance occur whiplash injuries QTF I°?
2. In which way affects the therapy recommendation “act as usual” the pain symptoms and the disability of patients with whiplash injuries QTF I°?

## 2. Method

### 2.1 Subjects

Between February 2009 and August 2011 470 patients with acute whiplash injuries had been treated in the emergency department at the University hospital of Ulm in Germany. The injuries had been classified corresponding to the QTF between the third and fifth day after the accident. The classification system of the QTF consists of four grades of severity (Spitzer et al., 1995). QTF I° correspond to patients with neck pain without musculoskeletal signs and free range of motion, QTF II° to patients with neck pain with musculoskeletal signs and restricted range of motion, QTF III° to patients with neurological symptoms and QTF IV° to patients with structural osseous or ligamentous injuries. Patients who had suffered previous injuries of the cervical spine or who had muscular, neurological or mental disorders were excluded from participation in the study. At the intake examination osseous injuries were excluded by appropriate radiographic imaging. Patient with QTF I° injuries were asked to participate at the study.

### 2.2 Study Procedure

All patients, who were enrolled in the study, gave their written informed consent to the study participation. Initially they got a standardised prescription for non-steroidal anti-inflammatory drugs (NSAID) with the recommendation to take the medication for ten days. The patients were asked to document the period of how long the painkillers were taken.

Three days after the whiplash trauma, patients’ pain score and disability score were determined and their range of motion in the cervical spine was assessed. In a detailed consultation session, the patients were explained the quality of injury without any gravity. The patients were given the recommendation to resume their usual activities without changing anything. A certificate of disability of maximal five days was only issued if the patient had occupational activity with a high level of physical exposure. No further therapeutic measures were recommended.

The patients were asked, to contact the study doctors if the symptoms last longer than two weeks. After six months the patients were contacted by phone. In patients with persisting symptoms the pain score and disability score were determined as well as their range of motion in the cervical spine was assessed again in a second examination.

### 2.3 Pain Score

Patients’ pain score was determined using two semi-quantitative visual analogue scales (VAS), each 10cm in length. Zero value represents no pain and value 10 represents maximal pain intensity. Pain scores below 0.4 can be considered as no pain, between 0.5 to 4.4 as mild pain, 4.5 to 7.4 as moderate pain and 7.5 to 10 as severe pain (Jensen, Chen, & Brugger, 2003). Patients were asked to indicate their average degree of pain and their most severe pain, respectively. The pain score was calculated as the average of these two values.

### 2.4 Pain Disability Index (PDI)

Patients’ disability score was determined on the basis of seven semi-quantitative VAS, each 10cm in length (Chibnall & Tait, 1994; Pollard, 1984). Zero value represents no deficit of ability and value 10 represents maximal disability. Patients reported their respective limitations in family life, recreation and sports, social activities, occupation, sexuality, personal tasks (dressing, shopping) and life-sustaining activities (eating, breathing). The PDI was calculated as the median of the seven individual scores. Therefore the minimal index is 0 and the maximal index is 70. The higher the index the greater is the person’s disability due to pain.

### 2.5 Analysis

Data were evaluated descriptively. Results were tested for statistical significance using the Wilcoxon test for paired samples. The percentage rate of persisting pain was determined using the results obtained at six-month follow-up.

## 3. Results

### 3.1 Subject Participation

Out of the 470 patients (283 women and 187 men) between 18 and 68 years (median: 45 years) with acute whiplash injuries 359 patients (76.4%) were classified as QTF I°, 75 patients (16.0%) as QTF II°, 18 patients (3.8%) as QTF III° and 18 patients (3.8%) as QTF IV°. 162 patients with QTF I° whiplash injuries of the cervical spine (patients with neck pain without musculoskeletal signs) and free range of motion could be investigated in this study.

### 3.2 Pain Score

**Figure 1:**
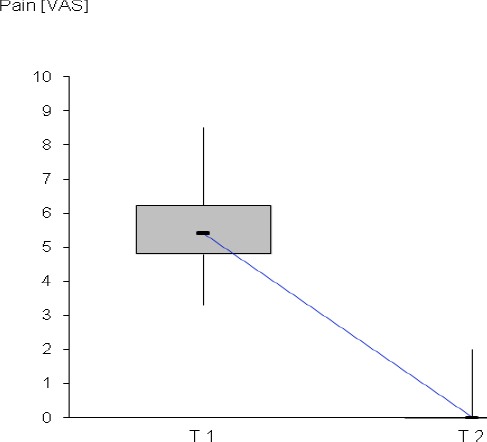
Pain score after injury (T 1) and after six months (T 2) VAS –visual analogue scale, horizontal bar – median, box – 1st quartile and 3rd quartile, vertical bars – maximum and minimum

After injury the median pain score assessed by VAS was 5.4 (min = 3.3; max = 8.5). In the further study course no patient used the telephone hotline to complain persisting pain. After six months 5 of the 162 patients interviewed by phone complained intermittent pain symptoms with VAS values < 2 (see Figure 1). This is consistent with a chronification rate of 3.1%.

### 3.3 Pain Disability Index

**Figure 2:**
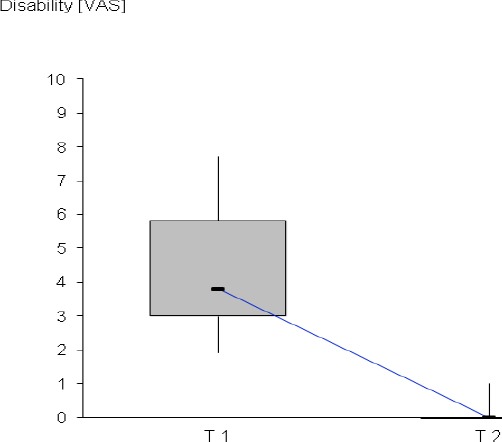
Disability score after injury (T 1) and after six moths (T 2) VAS –visual analogue scale, horizontal bar – median, box – 1st quartile and 3rd quartile, vertical bars – maximum and minimum

After injury, the median PDI was 3.9 (min = 1.9; max = 7.7). After six months 3 of the 162 patients interviewed by phone stated persisting disability during sporting and physical activities. The disability score was < 1 in all of the three patients (see Figure 2).

## 4. Discussion

The results of this study show that the occurrence of so-called minimal whiplash injuries (QTF I°) with a percentage of 76.4% is relatively high. In this patient collective the initial pain treatment and the therapy recommendation “act as usual” led to a satisfying healing course with a quite low chronification rate of 3.1%.

Up to now the therapeutic recommendations for both patient collectives (QTF I° and QTF II°) has been the same (Peeters et al., 2001; Scholten-Peeters et al., 2002; Spitzer et al., 1995). The initial therapeutic measures consisted of NSAID, the immobilization of the cervical spine in a soft-collar and the formulation of physical therapy. The following main therapy principles could be identified in the literature:

1. “act as usual”
2. “passive” physiotherapy
3. “active”physiotherapy
4. Multimodal therapy (= “active” and “passive” physiotherapy)

As already stated in the introduction section up to now only mixed populations with QTF I° and QTF II° patients has been investigated. This fact influences not only the prognosis but also the necessity of an appropriate therapy plan. As the QTF I° patients only complaint pain symptoms without a functional deficit therapeutic measures, which are aimed to protect or restore structural limitations, are not justified. The mainly therapeutic goals for these patients should be the adequate pain treatment and the quickest possible reestablishment of a complete analgesia.

In this study – under strict selection of the appropriate patients – the therapy recommendation “act as usual”, which guarantees a cheap and simple therapy option, has been evaluated. The principle of the therapy recommendation “act as usual” is based on the assumption that the sustained injury is self-limiting. The goal of that is the quickest possible socio-professional reintegration of the patients.

Secondary benefits of being sick, the attention given by medical staff, the taking advantage of work incapacity, the speculation of legal proceedings and the mental expectation of further diagnostic or therapeutic measures are discussed as possible reasons for the occurrence of pain chronification (Ferrari, 2000; Sullivan, Adams, Martel, Scott, & Wideman, 2011). Therefore the arising feeling of being sick should be avoided by the appeasing invitation to perform all “normal” activities of the daily live without any health risk (Borchgrevink et al., 1998). May be the acceptation of responsibility concerning the own healing leads to an increase of motivation and a higher willingness to tolerate possible short-term episodes of pain symptoms (Sterling, Carroll, Kasch, Kamper, & Stemper, 2011).

The results of this study – investigating isolated patients with QTF I° injuries – show, that under the condition of an appropriate selection of the patients the usage of the therapy recommendation “act as usual” leads to very good long-term results. After six months only 5 of 162 patients (3.1%) interviewed by phone complained intermittent pain symptoms. Obviously the pain intensity was low enough in all patients as none of the patients used the telephone hotline to complain persisting pain symptoms and ask for further therapy measures. The general performance of physical therapy is under consideration of a chronification rate of 3.1% not justified.

In case of persisting pain symptoms longer than two weeks and the possibly occurrence of associated functional deficits the therapy concept should be changed and adapted to the new situation. In this context additional active physical therapy measures seems quite meaningful. Concerning the healing prognosis the delay of two weeks until beginning physical therapy could be neglected in these patients as active physical measures relevant for healing normally could start at the earliest after 10 days. To get the general flexibility of changing therapy concepts a detailed and comprehensive initial consultation of the patient about the injury severity and the predicted healing process and possible complication seems to be very helpful.

If the differentiation between patients with QTF I° and QTF II° injuries is not or not accurately performed, one has to fear that the general therapy recommendation “act as usual” leads to an increase of the chronification rate. In a therapy study with a mixed patient collective of QTF I° and II° patients, Borchgrevink et al. (1998) compared two-week immobilisation with a cervical collar followed by the therapy recommendation “act as usual” with the immediate therapy recommendation “act as usual”. He could indeed show that the isolated therapy recommendation “act as usual” led to a significant better long-time outcome. Nevertheless after six months the chronification rate in this group was about 10%. Hartling et al. (2001) described in a retrospective analysis of QTF I° patients a chronification rate after six months of 30%. It is conceivable that a poor documentation of clinical findings like deficits of range of motion and musculoskeletal signs leads to a false classification of QTF II° patients as QTF I°.

In the case of Germany, in which the study has been performed, the orienting economic analysis leads to following results. Under the assumption of ordering 18 therapy units on average of passive physiotherapy (23.60€) or active physiotherapy (42.40€) the therapy costs per patient add up to 424.80€ or 763.20€. In comparison to that the therapy recommendation “act as usual” causes no therapy costs. In Germany 2011 306.266 motor-vehicle related accidents with 323.380 minor injuries were registered (Statistisches_Bundesamt_Wiesbaden, 2012). A detailed analysis concerning the exact percentage rate of cervical spine injuries has not been performed, but expert opinions suggest a percentage rate of about 60% (Keidel, 2000). Under assumption of an incidence rate of 200.000 whiplash injuries per year in Germany and based on the detected frequency rate of QTF I° patients of 76.4%, 152.800 QTF I° patients lead to huge overall therapy costs of 64.909.440€ (passive physiotherapy) or 117.717.120€ (active physiotherapy). Not considered in this exemplarily calculation are costs resulting from work incapacity, financial compensations and legal proceedings, which could be probably also reduced.

Under consideration of the high frequency of QTF I° injuries in comparison to the total number of whiplash injuries – in this study 76.4% (359 out of 470) patients – the therapy recommendation “act as usual” in combination with an adapted pain treatment offers a huge potential of costs reduction. Nevertheless the therapy recommendation “act as usual” has up to now not been sufficiently established in the health systems for the treatment of QTF I° whiplash injuries. May be multi-centre studies assessing the socio-economic effects in the health systems and confirming the results of the exemplary economic analysis mentioned-above should be performed. Independently of that the knowledge of optimizing the treatment guidelines for QTF I° patients should be expanded into the general medicine of the health systems by improving the education of the physicians and therapists.

## 5. Conclusions

General therapy recommendations do not cope with the specific therapeutic needs of mixed patient collectives after whiplash injuries. Based on medical and socio-economic reasons physical therapy should be formulated dependent of the severity of the sustained injury. Normally patients with whiplash injuries QTF I° do not need physical therapy. The therapy recommendation “act as usual” in combination with an adapted pain treatment is sufficient. An escalation of therapy measures should be reserved to patients with complicated healing processes.
